# Primerdiffer: a python command-line module for large-scale primer design in haplotype genotyping

**DOI:** 10.1093/bioinformatics/btad188

**Published:** 2023-04-17

**Authors:** Xiaoliang Ren, Yanwen Shao, Yiwen Zhang, Ying Ni, Yu Bi, Runsheng Li

**Affiliations:** Laboratory of Marine Organism Taxonomy and Phylogeny, Qingdao Key Laboratory of Marine Biodiversity and Conservation, Institute of Oceanology, Chinese Academy of Sciences, Qingdao 266071, China; Department of Infectious Diseases and Public Health, Jockey Club College of Veterinary Medicine and Life Sciences, City University of Hong Kong, Hong Kong, China; Department of Infectious Diseases and Public Health, Jockey Club College of Veterinary Medicine and Life Sciences, City University of Hong Kong, Hong Kong, China; Department of Biomedical Sciences and Tung Biomedical Sciences Centre, City University of Hong Kong, Hong Kong, China; Department of Infectious Diseases and Public Health, Jockey Club College of Veterinary Medicine and Life Sciences, City University of Hong Kong, Hong Kong, China; Department of Infectious Diseases and Public Health, Jockey Club College of Veterinary Medicine and Life Sciences, City University of Hong Kong, Hong Kong, China; Southern Marine Science and Engineering Guangdong Laboratory (Guangzhou), Guangzhou, China

## Abstract

**Motivation:**

Primer design is a routine practice for modern molecular biology labs. Bioinformatics tools like primer3 and primer-blast have standardized the primer design for a specific region. However, large-scale primer design, especially for genome-wide screening, is still a labor-intensive job for most wet-lab researchers using these pipelines.

**Results:**

Here, we present the primerdiffer pipeline, which can be used to batch design primers that differentiate haplotypes on a large scale with precise false priming checking. This command-line interface (CLI) pipeline includes greedy primer search, local and global *in silico* PCR-based false priming checking, and automated best primer selection. The local CLI application provides flexibility to design primers with the user’s own genome sequences and specific parameters. Some species-specific primers designed to genotype the hybrid introgression strains from *Caenorhabditis briggsae* and *Caenorhabditis nigoni* have been validated using single-worm PCR. This pipeline provides the first CLI-based large-scale primer design tool to differentiate haplotypes in any targeted region.

**Availability and implementation:**

The open-source python modules are available at github (https://github.com/runsheng/primerdiffer, https://github.com/runsheng/primervcf) and Python package index (https://pypi.org/project/primerdiffer/, https://pypi.org/project/primervcf/).

## 1 Introduction

PCR is still the dominating method to determine if a certain sequence is presented in a sample or not. Bioinformatics tools like primer3 (Untergasser et al. 2012) and primer-blast (Ye et al. 2012) have standardized the primer design for a specific region. However, large-scale primer design, especially for genome-wide screening, is still a labor-intensive job for most wet-lab researchers using these pipelines. Some web applications have been developed to design large-scale primers for real-time quantitative PCR (Arvidsson et al. 2008; Jeon et al. 2019) or pre-curated short sequences (You et al. 2008; Ramirez-Gonzalez et al. 2015). Still, none are suitable to design primers for chromosomal scale genotyping.

For evolutionary biologists working on intra- or inter-species hybridization, a routine task could be genotyping the chromosomal crossover between haplotypes. For this purpose, the haplotype-specific primers for given syntenic regions would be needed to get the introgression boundaries. However, most of the web-based tools are specialized for model species and lack the feasibility for user-specific genomes. A command-line interface (CLI)-based pipeline would be more suitable for intermediate users who need more flexibility on target sequences.

Here, we present the primerdiffer pipeline, which is used to batch design primers to differentiate haplotypes with precise false priming checking. We employed a greedy primer design method to walk alongside the genome with given intervals or indels. Each primer is validated by *in silico* PCR to ensure specificity.

## 2 Implementation and design

The primerdiffer pipeline is used to design haplotype-specific primers, which can only amplify one haplotype but not the others. These primers can thus detect if a given fragment from a specific haplotype is presented in your sample.

The full pipeline is divided into two parts, the primerdiffer module and the primervcf module, used to design primers for genotyping haplotypes with different similarities. The pipeline is invoked using a CLI written in python and requires a Unix-based operating system. The Python-abstracted API from the Primer3-py module is used to design primers by calling Primer3 libraries (Untergasser et al. 2012). The default parameters in the Primer3 library are inherited for the primer design. And the users can override the default cutoffs by providing additional lines in a user-defined config file. In addition, the primerdiffer module requires a local blast (Boratyn et al. 2012) for primer specificity check. The primervcf module is built on top of the primerdiffer module.

## 3 Usage

### 3.1 Design primers for inter-species haplotype genotyping

The inter-species haplotypes have low sequence similarity, and a greedy method can be used to pick primers. Generally, the genome assembly for each haplotype would be available. The primerdiffer module takes two reference genome files (genome1 and genome2) in FASTA format as input. Positional information for genome1 can be provided to define the region used to search genome1 unique primers. We built an *in silico* PCR function based on blast (Ye et al. 2012) to check if the primer can only amplify one unique region in genome1 and no regions in genome2.

**Figure 1:**
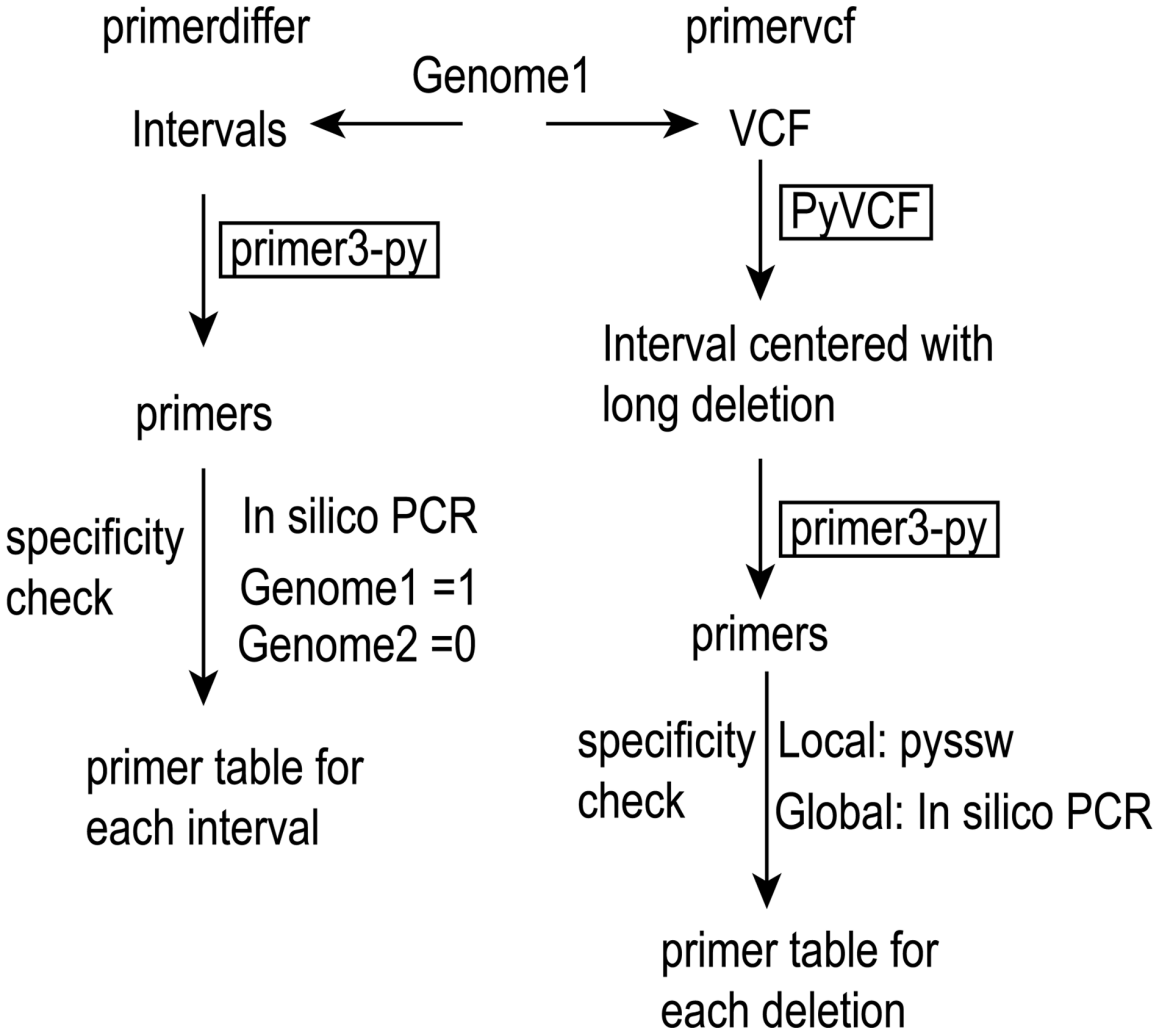
Implementation of the primerdiffer pipeline

By default, the given region would be divided into 5-kb intervals, and the pipeline would try to pick one primer for each interval (Fig. 1). For each given region, the primerdiffer pipeline will try to use the top five primers generated by the Primer3 library. Each primer would be checked by *in silico* PCR against genome1 and genome2 with a maximum product size of 2 kb. If one primer can pass the specificity check, then the primer will be the output for this region. The full output would be a series of primers that only amplify genome1 but not genome2. The primer targeting position, forward/reverse primer, and product size will be written to a file.

### 3.2 Design primers for intra-species haplotype genotyping

The intra-species haplotypes have high sequence similarity and can be represented by variations. And genome2 will not be available for specificity check. The deletions (≥10 bp by default) are extracted from the VCF files using the PyVCF (https://pypi.org/project/PyVCF3/) module. By forcing the overlapping of the forward or reverse primer with the deletion region, the haplotype-specific primers can be generated.

Due to the repetitive nature of sequences near most deletions, both local and global primer specificity checks will be applied. The global specificity check is similar to the primerdiffer module, wherein the only primers retained are the ones that amplify a unique region in genome1. For the local specificity check, both the original sequence from genome1 and the modified sequence by removing the deleted nucleotides representing the other haplotype are used as references. The Striped Smith–Waterman (SSW) alignment (Zhao et al. 2013) is used to check if the primer overlaps with the new junction sequences (Fig. 1). We have modified the python wrapper for SSW alignment and maintained a minimal functional python module in PyPI (https://pypi.org/project/pyssw/) for easier installation.

### 3.3 Additional tools

The primerdeign.py script can also be used as a general-purpose primer design CLI tool by adjusting the config file. The ispcr.py script can be used as a CLI tool for *in silico* PCR with given primers and references. The fq2vcf.py script can generate the VCF file by mapping NGS reads to the reference with the bwa-mem (Li 2013).

### 3.4 Example results

Four pairs of species-specific primers designed to genotype *Caenorhabditis briggsae* or *Caenorhabditis nigoni* X chromosome are picked and validated using single-worm PCR (Supplementary Fig. S1). All eight primers showed specific amplification for their targets.

## Supplementary Material

btad188_Supplementary_Data
