# Supplementary material for: Primerdiffer: a python command-line module for large-scale primer design in haplotype genotyping
Source: Bioinformatics. 2023 Apr 17;39(4):btad188. doi: 10.1093/bioinformatics/btad188 (PMC10121334; doi:10.1093/bioinformatics/btad188)

A

C. briggsae specific primers

1. CbrX:14226211-14226498	AAAGCAGGACTTGGCAACAG	TGTGACACTTCTGGCTTTCG	288
2. CbrX:15238998-15239524	GCAAACGACCTTTCTTGAGC	CCATCTTTGACATTCCGAAAA	527
3. CbrX:15524325-15524652	CCCCACATTGTTGGAAAGTC	TTGTCAGGTGCAGATGTGGT	328
4. CbrX:16621007-16621362	AGCCAACAGACTCTCGCAAT	AGGGCAAACAATTCAGATGG	346

C. nigoni specific primers

5. CniX:17908135-17908445	AGTGTCAGCTCCAACTCAAA	ACCTCCATTGCATCTATGGG	311
6. CniX:18450156-18450461	GAATTCCGCAACCGAGAAC	CAAAGGCTTAAGCGGAACAA	306
7. CniX:18605081-18605481	TCGCAAATCGAAAAATCGGT	GAGGACCACAAAAAGAACGG	401
8. CniX:20550095-20550408	CACTGGCATCACATTTCTCG	CTCCCCAACAAAAACGGTTT	314

B

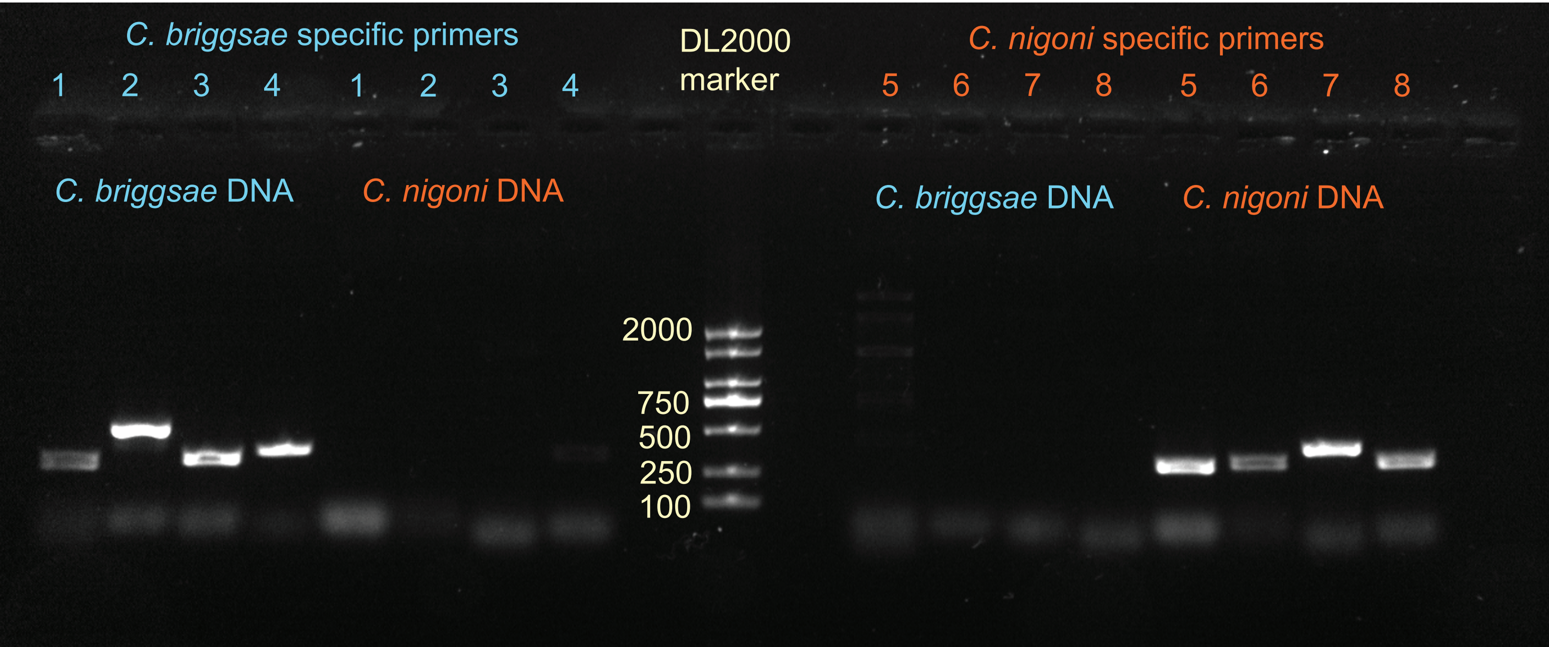

Supplement: btad188_Supplementary_Data [file btad188_supplementary_data.pdf]
